# Taming the Tiger: Partial Input Block Results in a Stable, Organized Coronary Sinus Activation During Atrial Fibrillation

**DOI:** 10.19102/icrm.2024.15045

**Published:** 2024-04-15

**Authors:** Emir Baskovski, Timucin Altin, Omer Akyurek

**Affiliations:** 1 Department of Cardiology, Faculty of Medicine, Ankara University, Ankara, Turkey

**Keywords:** Atrial fibrillation, atrial mapping, atrial tachycardia

## Abstract

In this manuscript, we present a case where coronary sinus activation was organized and stable despite the rhythm being atrial fibrillation. We discuss the possible mechanisms of this rare occurrence.

A 65-year-old woman with a history of atrial fibrillation (AF) was admitted to undergo AF ablation following an early recurrence after cardioversion. The patient was informed about the study procedure and provided written consent.

A decapolar catheter (WEBSTER™ CS; Biosense Webster, Diamond Bar, CA, USA), placed inside the coronary sinus (CS), depicted an organized activity with the cycle length varying between 190 and 200 ms and a distal-to-proximal activation sequence. Then, the left atrium (LA) was mapped using the Pentaray (Biosense Webster) catheter. On the voltage map, with the upper and lower thresholds set to 0.3 and 0.1 mV, respectively, large low-voltage areas were observed at the posterior and anteroseptal walls **(Figure 1)**. The activation map was uninterpretable, similar to an activation map that can be obtained during AF **(Figure 1)**. Local electrograms obtained at both the anterior and posterior sites depicted AF-like chaotic activity. An organized activity within the LA was in the area between the left atrial appendage and the mitral isthmus. Pulmonary vein isolation, posterior wall isolation, and anterior mitral line (utilizing a SMARTTOUCH SF catheter; Biosense Webster) aiming to ablate the complex fractionated atrial electrograms resulted in a slight change of CS activation, upon which the LA was remapped. An organized activation of LA consistent with atrial tachycardia was observed with very long fractionated electrograms, which were thought to be the critical component, in the vicinity of the anterior mitral line. Ablation at this location terminated the tachycardia. During the sinus rhythm, a mitral isthmus block was not present; however, there was a block across the anterior mitral line. The case was completed after cavotricuspid isthmus ablation. The tachycardia was non-inducible via pacing maneuvers at the end of the procedure. Three months after the procedure, the patient was still in sinus rhythm.

The CS musculature plays one of the vital roles in the initiation and perpetuation of AF.^1^ Focal organized activity in the LA during AF is a common occurrence; however, the CS is almost universally chaotic. Electroanatomically, the CS is connected to the LA via myocardial sleeves and the vein of Marshall, both of which have musculature that is thought to be arrhythmogenic.^2,3^ During AF, multiple waveform collisions result in a commonly observed chaotic activation of the CS. In the presented case, an input block in one or more of the CS connections, resulting either from the functional input block, multiple constant lines of blocks in the fibrotic LA, or most likely a combination of both, leads to seemingly organized CS activity that cannot be distinguished from an atrial tachycardia activation^3,4^ **(Figure 2)**. A careful inspection of the activation map shows that the CS is activated via connections with the left ridge and/or the vein of Marshall, as there is no input from the more proximal (CS connections to the posterior left atrial wall and right atrium) areas to the CS **(Figure 2)**. Ablation of multiple complex fractionated atrial electrograms terminated the AF and led to an atrial tachycardia, which had a critical component in the anterior wall. No mitral isthmus block could be demonstrated after termination of the tachycardia, implying the functional nature of the block during AF.

In conclusion, a functional block of CS connections as well as multiple lines of block in the LA may lead to a slow and organized CS activation during AF that cannot be distinguished from CS activation during an atrial tachycardia.

## Figures and Tables

**Figure 1:**
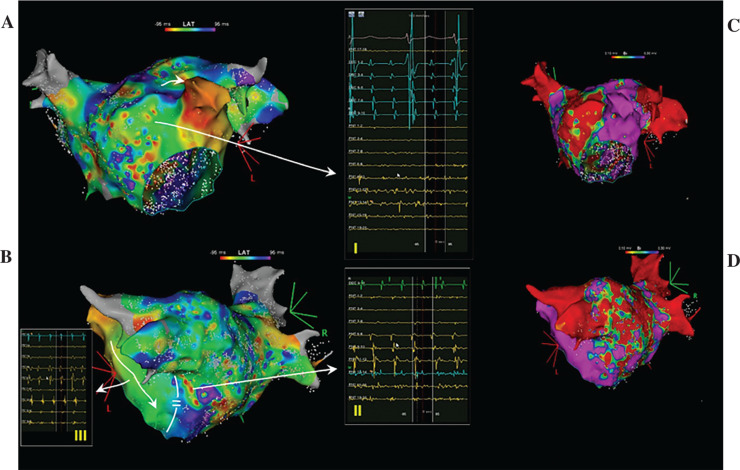
Electroanatomic map of the left atrium obtained during atrial fibrillation. **A and B:** An activation map, which is uninterpretable despite having a stable window of interest based on very organized and stable activation of the CS, is depicted. Insets depict the chaotic activation in the anterior (inset I) and posterior (insert II) walls as well as very organized activation of the left ridge (inset III), which was probably possible due to a functional block at the mitral isthmus (shown on **B**). **C and D:** Large low-voltage areas in the left atrium with the upper and lower thresholds set at 0.3 and 0.1 mV, respectively, are depicted.

**Figure 2:**
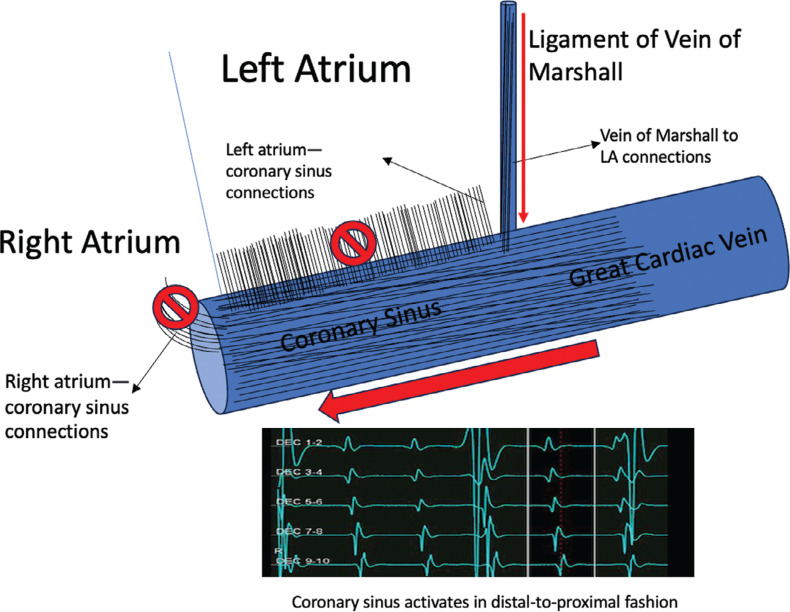
A schematic representation of various myocardial connections to the coronary sinus (CS). The CS is covered by a myocardial coat, which has a connection to the right atrium, posterior left atrium (LA), and the anterior ridge of the LA via the vein of Marshall. We hypothesized that the functional block in the right atrial and myocardial coat extending to the posterior LA render the vein of Marshall connection the sole input to the CS; thus, activation wavefront proceeds from distal to proximal (DEC1–2: distal CS; DEC9–10: proximal CS; DEC3–4, 5–6, 7–8: middle three electrode pairs). Therefore, the usual chaotic activation during atrial fibrillation, caused by multiple inputs, is replaced by a fairly stable activation originating from a single source.
